# Schwann cells promote the migration and invasion of colorectal cancer cells *via* the activated NF-κB/IL-8 axis in the tumor microenvironment

**DOI:** 10.3389/fonc.2022.1026670

**Published:** 2022-11-17

**Authors:** Shuhai Chen, Mingyou Chen

**Affiliations:** ^1^ Department of Digestive and Transplant Surgery, Graduate School of Biomedical Sciences, Tokushima University, Tokushima, Japan; ^2^ Department of Cardiology, The First Affiliated Hospital of Shandong First Medical University & Shandong Provincial Qianfoshan Hospital, Shandong Medicine and Health Key Laboratory of Cardiac Electrophysiology and Arrhythmia, Jinan, Shandong, China

**Keywords:** Schwann cell, tumor microenvironment, colorectal cancer, perineural invasion, interleukin-8

## Abstract

**Background:**

Evidence has shown neurons and glial cells were closely related to tumor progression. As the predominant glial cells in the external innervated nerves of the gastrointestinal, the role of Schwann cells (SCs) in colorectal cancer (CRC) has not been well explored.

**Methods:**

HCT-116 and HT-29 CRC cells were treated with conditioned medium (CM) from SCs, and the cells’ proliferative and migrating capacities were examined. Cytokine array analysis was used to identify the tumor-promoting-cytokines from SCs-CM. Molecular changes from SCs after being co-cultured with tumor cells were detected by ELISA and reverse transcription-quantitative PCR. The activation of the nuclear factor kappa B (NF-κB) signaling pathway in SCs was demonstrated by immunofluorescence staining. Neutralizing antibody was used to verify the tumor-promoting effects of key cytokine.

**Results:**

Migration and invasion of CRC cells were markedly aided by CM from SCs *in vitro*. Interleukin-8 (IL-8) was identified as an effective factor. SCs co-cultured with CRC cells upregulated IL-8 expression, which may be related to its activated NF-κB signaling pathway. Neutralization of IL-8 attenuated the tumor-promoting effect of SCs.

**Conclusion:**

The present study identified a new mechanism of tumor-neuroglia interaction, enriching the concept of the tumor-neural axis in the tumor microenvironment of CRC, which also inspired potential targets for anti-cancer therapies.

## Introduction

As one of the most common malignancies, colorectal cancer (CRC) is responsible for a great number of cancer-related deaths worldwide (1). In addition to having a high risk of metastatic spread through blood and lymphatic vessels, CRC shows a distinctive characteristic of nerve dependence (2), which leads to a 15.7%-38.9% occurrence of perineural invasion (PNI) (3). It has been widely reported that the presence of PNI would indicate a more aggressive clinicopathological profile of CRC and lead to a poor prognosis (3–5).

The gut is a highly innervated organ that is both innervated by the peripheral nervous system (PNS) including sympathetic, parasympathetic and sensory branches from outside, and intrinsically regulated by the enteric nervous system (6). Valès S et al. previously revealed an interaction between CRC cells and enteric glial cells (EGCs). The EGCs, once activated by tumors, acquired a pro-tumorigenic phenotype and stimulated cancer stem cells-driven tumorigenesis through a prostaglandin E2/EP4/epithelial growth factor receptor-dependent pathway (7). Schwann cells (SCs), the most abundant glial cell type in the PNS, are present in almost every anatomical part of the body (8, 9). Unlike enteric glia, SCs are often considered to be associated with the external innervation of gut. Nevertheless, the markers used for enteric glia, like Sox10, S-100β, glial fibrillary acidic protein, can also be expressed by SCs, making it challenging to differentiate (6, 10). SCs were investigated in the precancerous stages of pancreatic and colon cancers, suggesting their specific affinity for cancer cells (11). Moreover, recent emerging insights indicated that, besides their functions in PNI (3, 12), SCs could also promote tumor progression by acting directly on tumor cells (13–15) or interacting with other components of the tumor microenvironment (TME) (16, 17). However, the role of the SCs in the development and progression of CRC remains poorly recognized. In this study, we found that in the TME of CRC, the nuclear factor kappa B (NF-κB) signaling pathway in SCs was activated by tumor cells. SCs could promote the migration and invasion of CRC cells by secreting more interleukin-8 (IL-8), which in turn promoted the tumor progression. Overall, we provided a new understanding of tumor-neuroglia interaction, and enriched the concept of the tumor-neural axis in the TME of CRC, which might provide targets for anti-cancer therapies.

## Materials and methods

### Cell culture

Human colorectal cancer cell lines HCT−116 (ECACC 91091005) and HT−29 (ECACC 91072201) were supplied by The European Collection of Authenticated Cell Cultures (ECACC). Human SC line NF1 ipNF95.6 (ATCC^®^CRL-3389™) was obtained from American Type Culture Collection (ATCC). The Dulbecco’s Modified Eagle Medium (DMEM, Thermo Fisher Scientific, Inc.) supplemented with 10% fetal bovine serum (FBS, Thermo Fisher Scientific, Inc.) and 1% penicillin-streptomycin (Thermo Fisher Scientific, Inc.) was utilized to cultivate all of the cell lines in this study according to the previous description (15, 18). Cells were cultured at 37°C in 5% CO2 and were chosen for experiments in mycoplasma-free conditions during the logarithmic growth phase.

### Conditioned medium collection and cell co-culture

Complete media was used to grow the cells until they were around 80% confluent. The cells were twice washed in pre-warmed PBS, before being incubated in new DMEM without serum. After 48 hours of incubation, the supernatant was collected and centrifuged (500 × g) for 20 minutes at room temperature, and then filtered through a 0.2−µm filter to remove the cell debris. The CM was kept at -80˚C and avoided frequent freezing and thawing referring to previously reported methods (19). To establish an *in vitro* co-culture system between tumor cells and SCs (15, 20), 1 × 10^5^ SCs were seeded at the bottom of six-well plates, while 3 × 10^5^ tumor cells (HCT-116 or HT-29) were added into the upper Transwell insert (0.4-µm pores, 353090; Corning, Inc.). The co-culture system was maintained with complete DMEM for 48h. Then, the cancer cells and the upper Transwell insert were removed from the co−culture system, and the remained monoculture of SCs named Schwann cell (Co-HCT-116) or Schwann cell (Co-HT-29), respectively. After that, fresh serum−free DMEM was changed for Schwann cell (Co-HCT-116) and Schwann cell (Co-HT-29) and the CM was collected in the same manner as above. For the IL-8 neutralization experiment, human IL-8/CXCL8 antibody (cat. no. MAB208-100, R&D Systems, Inc.) or normal human IgG control (cat. no. 1−001−A, R&D Systems, Inc.) was added to CM with a final concentration of 0.2 μg/mL according to the neutralization dose according to the product manual and incubated for 1 hour before use (21). A detailed flowchart gives a better explanation of the above process as a **Supplement Figure** (**Figure S1**)

### Cell proliferation assay

Cell proliferation assay referred to our previous research (22). HCT-116 or HT-29 cells were seeded at a density of 1×10^4^ cells/well into 96−well plates until adhesion. After PBS washing, the cells were cultivated with SC-CM. From days 1 to 3, the proliferative condition of the cells was monitored every 24 hours. According to the manufacturer’s instructions, fresh medium containing 10% volume of the cell counting kit-8 solution (Dojindo Molecular Technologies) was added to each group at each analysis time point. After 2 hours of incubation, the absorbance values at 450 nm were measured by a microplate reader (SpectraMax i3; Molecular Devices) to reflect the cell proliferation state.

### Wound healing assay

To generate a confluent monolayer at 90%, HCT-116 or HT-29 cells were seeded onto six-well plates at a density of 7×10^5^ cells per well and incubated overnight. Then, the middle of each well was then thoroughly scratched with a 200−µl pipette tip. The cells in each group were then cleaned with PBS before being cultured with the indicated CM for 36 hours without the addition of FBS according to the previous method (11). At 0 and 36 hours after scratching, the regions of the wounds were observed, and photographs were taken using a light microscope (magnification, ×40; DP22CU; Olympus). The ImageJ v1.46r software was used to compute the cell migration rates using the equation below: The relative migration rate is defined as [width (0 h)−width (36 h)]/width (0 h) ×100%.

### Migration and invasion assay

Migration and invasion assay referred to our previous research (19, 22). A 24-well transwell system (8.0−µm pores, Corning, Inc.) was used to achieve the migration and invasion assays. A 100−µl volume suspension containing a total of 5×10^4^/well serum-starved cancer cells per well was implanted into the upper chamber. After cell attachment, each group’s culture media was changed to the specified CM. In the lower chambers, the final FBS content was 10%, and in the upper chambers, it was 5%. The upper chambers were fixed with methanol and stained with 0.1% crystal violet after a period of incubation (24 h for the migration assay, and 36 h for the invasion assay), and non-migrated cells on the upper chambers were removed using a cotton swab. The residual cell count was then determined after photos were taken. For the invasion assays, the upper chambers of the transwell system were precoated with Matrigel (Corning, Inc.) overnight at 37°C.

### Cytokine array

A Human XL Cytokine Array kit (cat. no. ARY022B; R&D Systems, Inc.) was used to identify molecules in the CM from the SCs, HCT-116, and HT-29 cells. Briefly, the membrane containing 105 distinct capture antibodies was incubated overnight with indicated CM at 4˚C. On the next day, the membranes were incubated sequentially with the detection antibody cocktail and streptavidin-HRP after being rinsed with Wash Buffer. Next, the membranes were treated with a chemiluminescent detecting agent and exposed *via* an Amersham Imager 600 (Cytiva) to detect the signal intensities on the membranes. Each pair of positive dots represented the signals of highly expressed molecules and the luminescence intensity was quantified using ImageJ v1.46r software. The full list of all the antibodies is available in the product datasheet. The method for cytokine array referred to our previous study (22)

### Enzyme-linked immunosorbent assay

The Human IL−6 Quantikine ELISA kit (cat. no. D6050, R&D Systems Inc.) and Human IL−8 Quantikine ELISA kit (cat. no. D8000C, R&D Systems Inc.) were used to evaluating the concentrations of IL−6 and IL-8 in the CM according to previous report (23). The absorbance at 450 nm (with 540 nm serving as the correction wavelength) was determined using a microplate reader (SpectraMax i3; Molecular Devices). The relative secretion capacity was adjusted to account for the number of cells.

### Immunofluorescence staining

Cells were placed on chamber slides (cat. no. 191029, Matsunami), twice rinsed in PBS, and then fixed for 30 minutes at 4°C with 4% paraformaldehyde (163-20145; FUJIFILM). After being treated with 0.1% Triton X-100 (HFH10; Thermo Fisher Scientific) for 5 min to permeabilization, slides were blocked with 3% bovine serum albumin (BSA) for 60 min at room temperature. Then, an anti-NF-κB p65 antibody (ab16502, 1:500; Abcam) was used to incubate the slides at 4°C overnight as previous report (23). On the second day, after being washed with PBS for three times, the slides were incubated with Alexa Fluor 488-conjugated secondary antibodies (A32731, 1:1000; Thermo Fisher Scientific, Inc.) for 1 hour at 37°C in the dark. ProLong Gold Antifade Mountant with DAPI (P36931; Thermo Fisher Scientific) was used to stain the nuclei for 10 minutes in the dark following three PBS washes. Staining results were observed and recorded by a fluorescent microscope (BZ-X700; KEYENCE). BSA was used in place of the primary antibody for the negative controls. Using ImageJ v1.46r, the integrated density of immunofluorescence was calculated.

### Reverse transcription-quantitative PCR

Total RNA was extracted using an RNeasy Mini kit (Qiagen GmbH, Hilden, Germany) and the concentration of the collected RNA was determined using a spectrophotometer (NanoDrop™ 2000; Thermo Fisher Scientific). Subsequently, a High−Capacity cDNA Reverse Transcription kit (Applied Biosystems) was used to reverse transcribed 2.5 µg RNA into cDNA in a total 50 µl reaction system. Next, TaqMan qPCR was performed by a StepOnePlus Real−Time PCR system (Applied Biosystems) under the following reaction conditions: initial denaturation at 95°C for 3 min; 40 cycles of denaturation (95°C, 30 sec), annealing (58°C, 30 sec), and extension (72°C, 45 sec); final extension at 72°C for 10 min. The following TaqMan gene expression assays were used: IL-8 (assay ID, Hs00174103_m1, catalog, 4331182, FAM−labeled, Thermo Fisher Scientific); CCL2 (assay ID, Hs00234140_ m1, catalog, 4331182, FAM−labeled, Thermo Fisher Scientific). GAPDH (assay ID, Hs99999905_m1, catalog, 4326317E, VIC−labeled, Thermo Fisher Scientific) was used as the internal control to normalize the raw data. The results of the data analysis, which employed 2−ΔΔCq method (24), are shown as fold changes in the relative mRNA expression levels for each experimental group as compared to the control group.

### IL-8 gene and protein levels analysis in the public database

Box plots of the expression difference between colon adenocarcinoma (COAD) tissues and the corresponding normal tissues of the cancer genome atlas (TCGA) and the genotype-tissue expression (GTEx) database were obtained using the “Expression analysis-Box Plots” module of the gene expression profiling interactive analysis, version 2 (GEPIA2) web server (http://gepia2.cancer-pku.cn/#analysis), under the settings of P-value cutoff= 0.01, log_2_FC (Fold change) cutoff =1, and “Match TCGA normal and GTEx data”. We performed protein expression analysis of the clinical proteomic tumor analysis consortium (CPTAC) dataset using the UALCAN portal (http://ualcan.path.uab.edu/analysis-prot.html). Here, we investigated the amount of total IL-8 protein expression in primary colon cancer and the comparable normal tissues.

### Statistical analysis

All data are displayed as the mean ± SD. For the statistical analysis and making of the graphics, ImageJ v1.46r and GraphPad Prism v7.0 software (GraphPad Software) were used. For comparisons between two groups, the Mann-Whitney U test or unpaired Student’s t−test were applied. One-way analysis of variance (ANOVA) and Tukey’s *post hoc* test were used to assess differences between several groups. P<0.05 (two−sided) was regarded as statistically significant for any trials that involved more than three biological replicates.

## Results

### SCs promote the migration and invasion but not proliferation of CRC cells *in vitro*

**Figure 1:**
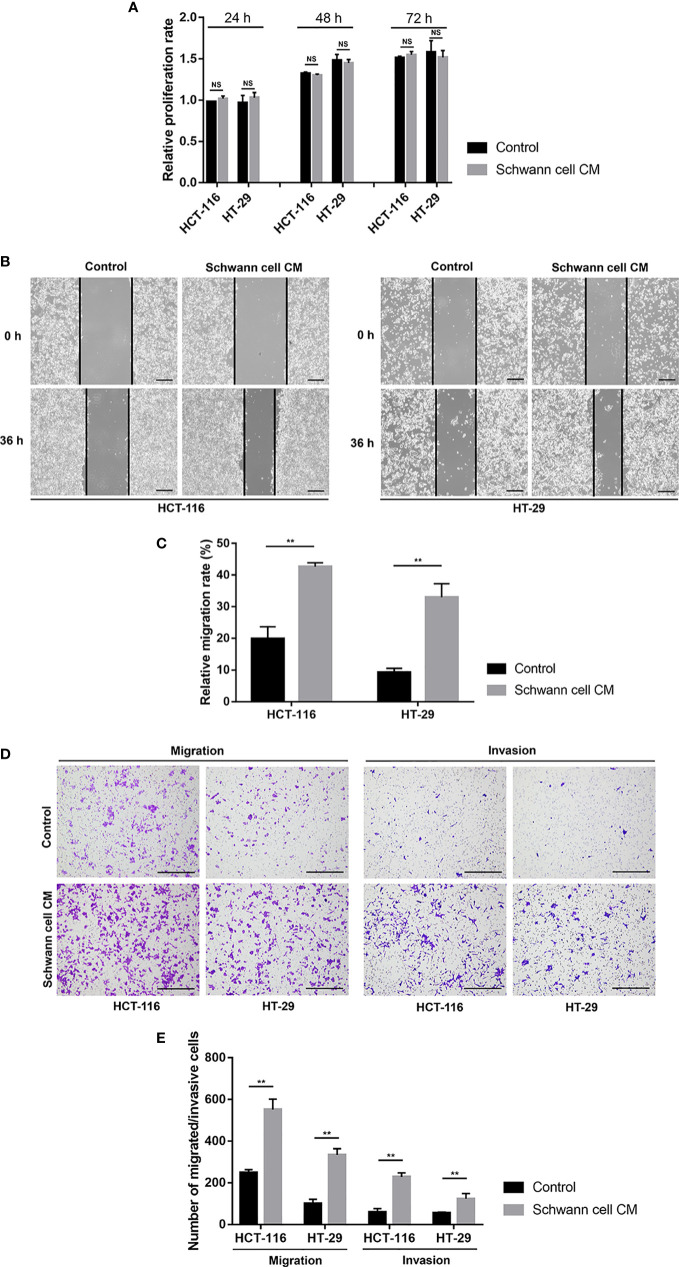
SCs promote the migration and invasion but not proliferation of CRC cells *in vitro*. **(A)** The findings of the cell counting kit-8 showed that SCs had no appreciable impact on the proliferation statuses of HCT-116 and HT-29 cells. **(B, C)** Wound healing (scale bar, 400 µm) suggested that SCs enhanced the migration of HCT-116 and HT-29 cells. **(D, E)** Transwell and Matrigel assays (scale bar, 400 µm) showed that SCs enhanced the migration and invasion of HCT-116 and HT-29 cells. **P<0.01. CRC, colorectal cancer; NS, not significant; SC, Schwann cell.

To investigate the effects of SCs on CRC cells, we used CM from SCs to incubate HCT-116 and HT-29 cells. As shown in **Figure 1A**, the proliferation of CRC cells was not changed significantly after SC-CM treatment after 24 h, 48 h and 72 h. However, the migration rates of HT-116 and HT-29 cells were significantly enhanced after SC-CM treatment as showed by the Would-healing assay (19.89 ± 3.77% vs. 42.67 ± 1.24% for HCT-116, 9.31 ± 1.24% vs. 32.97 ± 4.31% for HT-29; *p* < 0.01) (**Figures 1B, C**) and migration assay (**Figures 1D, E**). Moreover, the invasion capacity of both HCT-116 and HT-29 cells were also increased after SC-CM treatment as the numbers of invasive cells were highly increased (*p* < 0.01) (**Figures 1D, E**).

### IL-8 from SCs is attributed to promoting the malignant behaviors of CRC cells *in vitro*

**Figure 2:**
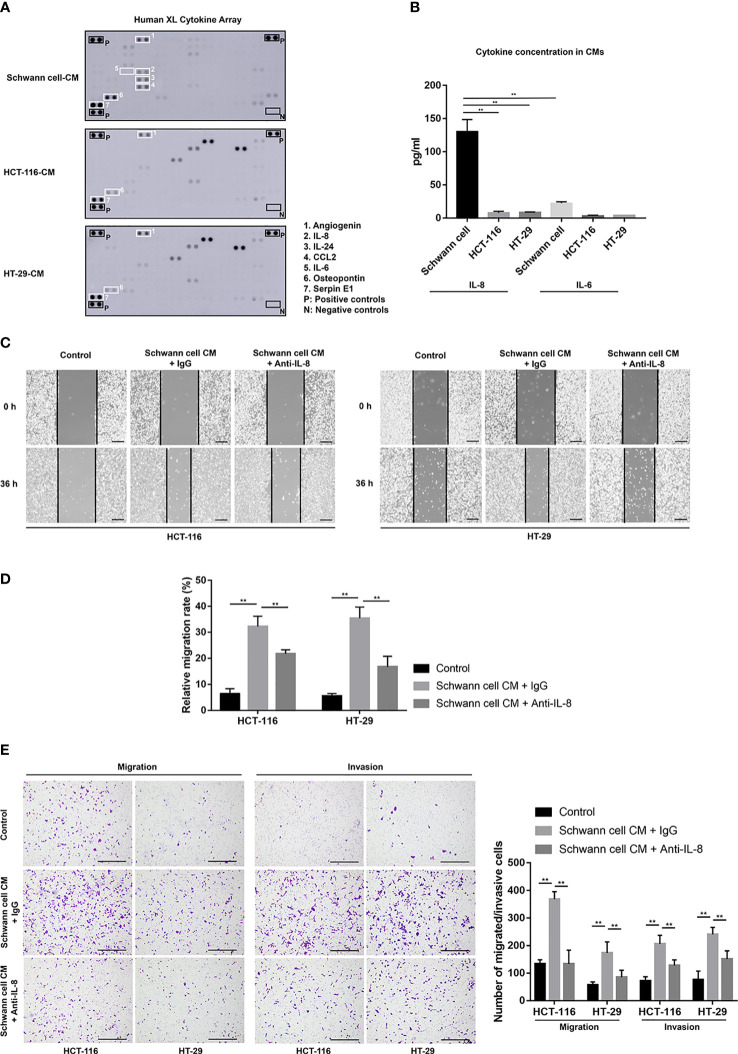
Anti-IL-8 treatment impairs the tumor-promoting effects of SCs on CRC cells. **(A)** A cytokine array’s original picture showed the paracrine factor profiles in SCs, HCT-116, and HT-29 cells. **(B)** ELISA results showed the secretion of IL-6 and IL-8 among SCs, HCT-116 and HT-29 cells. **(C, D)** Wound healing (scale bar, 400 µm) and **(E)** Transwell and Matrigel assays (scale bar, 400 µm) indicated that the neutralization of IL-8 in SC-CM suppressed the enhanced migration and invasion of HCT-116 and HT-29 cells. **P<0.01. CRC, colorectal cancer; CM, conditioned medium; IL-8, interleukin 8; SC, Schwann cell.

Since CM from SCs had the ability to promote migration and invasion. We inferred that soluble factors secreted by SCs might play this role. According to the cytokine assay results (**Figure 2A**), we could identify several cytokines that have a high amount of secretion in SCs. Among them, Angiogenin, Osteopontin and Serpin E1 were also highly secreted in HCT-116 and HT-29 cells, which means they could not take the unique role in the further enhancement of cancer malignancy by SCs. Previous reports showed that IL-6 and IL-8 played an important role in promoting tumor progression in TME. So we selected IL-6 and IL-8 as candidates. ELISA results confirmed that IL-8 had a high secretion only in SCs (130.66 ± 17.85 pg/ml) while not in HCT-116 (8.09 ± 2.38 pg/ml) and HT-29 cells (8.65 ± 0.65 pg/ml) (**Figure 2B**). However, IL-6 secretion was relatively low in both SCs and tumor cells. Based on these, we hypothesize that IL-8 from SCs could promote the malignancy of colorectal cancer cells *in vitro*. So we performed the Would-healing assay, migration assay and invasion assay after the neutralization of IL-8 in SC-CM. In SC-CM+Anti-IL-8 group, the migration rates of HT-116 or HT-29 cells were significantly decreased when compared with SC-CM group (21.75 ± 1.6% vs. 32.28 ± 3.93% for HCT-116, 16.79 ± 4.01% vs. 35.44 ± 4.28% for HT-29, *p* < 0.01) (**Figures 2C, D**). And consistent with our hypothesis, the promoting effects of SC-CM on the migration and invasion of CRC cells were reversed by the neutralization of IL-8 as shown by the migration and invasion assay (**Figure 2E**).

### NF-κB/IL-8 axis is activated in SCs after co-cultivation with CRC cells

**Figure 3:**
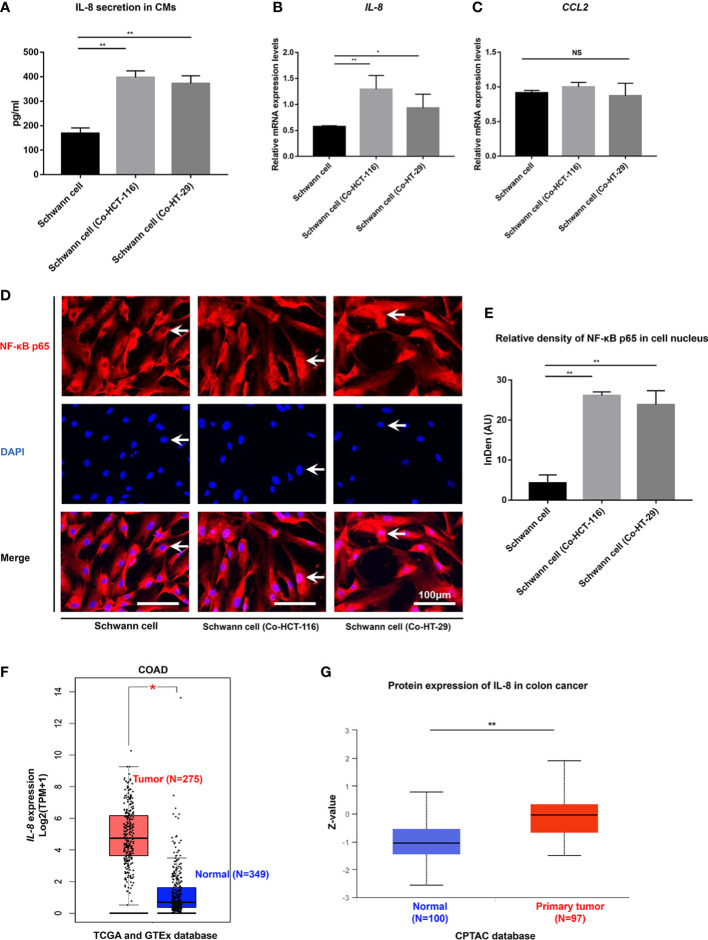
NF-κB/IL-8 signaling pathway is activated in SCs in the TME. **(A)** ELISA results showed the secretions of IL-8 from SCs were enhanced after co-cultivation with HCT-116 and HT-29 cells. Gene expression of IL-8 **(B)** and CCL2 **(C)** were detected using RT-qPCR in SCs and SCs after co-cultivation with HCT-116 and HT-29 cells. **(D)** Immunofluorescent staining results (scale bar, 100 µm) indicated the nuclear translocation of NF-κB in SCs after co-cultivation with HCT-116 and HT-29 cells. The arrows showed the nuclear expression of NF-κB p65 proteins. **(E)** Integrated density of NF-κB p65 in cell nucleus quantified in each group. **(F)** The gene expression levels of IL-8 in COAD in the TCGA project, the corresponding normal tissues of the GTEx database were included as controls. The box plot data were supplied. **(G)** The expression level of IL-8 total protein between normal tissue and primary tissue of colon cancer based on the CPTAC dataset. **P<0.01 *P<0.05. AU, arbitrary units; CM, conditioned medium; COAD, colon adenocarcinoma; CPTAC, clinical proteomic tumor analysis consortium; DAPI, 4,’6-diamidino-2-phenylindole, dihydrochloride; GTEx, genotype-tissue expression; IL-8, interleukin 8; NF-κB, nuclear factor kappa B; NS, not significant; SC, Schwann cell; TCGA, the cancer genome atlas.

To further explore the interaction between SCs and CRC in the TME, We co-cultivated SCs with HCT-116 and HT-29 cells. RT-PCR results showed that the gene expressions of IL-8 were upregulated in SCs after being co-cultured with HCT-116 cells or HT-29 cells (**Figure 3A**). The secretions of IL-8 in the CMs were also upregulated in SCs after being co-cultured with HCT-116 cells or HT-29 cells as demonstrated by ELISA results (168.96 ± 22.17 pg/ml vs. 396.8 ± 27.24 pg/ml vs. 372.47 ± 31.5 pg/ml, *p* < 0.01) (**Figures 3A, B**). We also checked the mRNA levels of CCL2 in SCs, which was also secreted by SCs. However, the gene expression of CCL2 was not changed significantly (*p* > 0.05) (**Figure 3C**). To further explore the mechanism of upregulation on IL-8 of SCs in the TME, we checked the NF-κB signaling pathway, which is the most classic regulation pathway of IL-8. The immunofluorescent staining results showed that the relative density of NF-κB p65 nuclear expressions were notably stronger in Schwann cell (Co-HCT-116) or Schwann cell (Co-HT-29) compared with SCs (*p* < 0.01) (**Figures 3D, E**), which indicated the activation of the NF-κB signaling pathway in SCs after being co-cultured with CRC cells. Finally, we acquired the gene (**Figure 3F**) and protein (**Figure 3G**) expressions of IL-8 in CRC tissue from the public database. Compared with normal tissue, higher expression of IL-8 was funded in the tumor area, which implied the enrichment of IL-8 in the TME.

## Discussion

In the present study, we focused on the role of SCs in the TME of CRC, which are glial cells from external innervated nerves of the gastrointestinal. Specifically, the promotion of migration and invasion of CRC cells by IL-8 from SCs was confirmed. Meanwhile, the NF-κB signaling pathway in SCs was activated after being co-cultured with CRC cells, which further increased its secretion of IL-8. This tumor-glial cell interaction exacerbated the tumor progression.

Recently, SCs have been regarded as active players of the TME (16). In addition to its widely recognized role in facilitating PNI, several studies have shown that several factors from SCs, such as CCL2 (13), CXCL5 (14), IL-6 (15) could directly promote the malignant phenotype of tumors. Based on our data, SCs could not affect the proliferation of CRC cells *in vitro*. However, the CM from SCs alone significantly promoted the migration and invasion capacities of CRC cells *in vitro*, while neutralizing IL-8 attenuated this effect. This demonstrated that IL-8 is at least one of the key factors in the SCs-CM to promote the progression of CRC. Different from the data from Su D et al. (15), the secretion of IL-6 from SCs in our results was not appreciable, which may be related to the difference in tumor types. Furthermore, it cannot be excluded that other factors may also play a role, such as CCL2, but the secretion of IL-8 from SCs was significantly increased upon activation by tumors, whereas CCL2 was not. Thus, this causal bidirectional molecular crosstalk would be more meaningful in the conceptual framework of TME.

According to our results, the gene expression and secretion of IL-8 were upregulated in SCs after being co-cultured with CRC cells. In fact, it is well known that tumor cells could “educate” surrounding cells through their direct or paracrine effects to form a microenvironment conducive to their own progression (19, 22). Recently, a study by Su D et al. (15) demonstrated that IL-1β secreted from pancreatic ductal adenocarcinoma cells led to aberrant activation of the NF-κB pathway in SCs in the interaction between pancreatic cancer and SCs. Indeed, SCs are remarkable in their ability to naturally (adaptively) reprogram and reprogram the surrounding environment (25). In our study, the classical inflammatory pathway NF-κB is activated in SCs among the TME of CRC and induced the enrichment of IL-8.

Our results emphasized the crucial role of IL-8 from SCs in the TME of CRC, so it might be an anti-tumor target to block the tumor-neuroglia interaction. Reparixin, a clinical-grade CXCR1/2 inhibitor, was reported to reduce the malignant features of human pancreatic cancer cells *via* inhibiting IL-8-CXCR1/2 signaling (26) In preclinical colon cancer models, the combination of the CXCR1/2 small molecule antagonist SCH-527123 and oxaliplatin resulted in greater reductions in cell proliferation, tumor growth, apoptosis, and angiogenesis than in monotherapy (27). However, considerable research is still needed before the predictive, prognostic and therapeutic value of IL-8 signaling in human cancers can be practically applied.

Admittedly, the lack of further evaluation of SCs and IL-8 expression in clinical specimens is a limitation of this study. However, a crucial challenge is that currently, markers for SCs and glial cells in the intrinsic enteric nervous system are not specific, which leads the difficulties in distinguishing (10). Moreover, this study focused only on the direct role of SCs and tumor cells. Further studies using multicellular models including neurons will be very useful for further investigating the role of glial cells in the development of PNI in CRC.

## Conclusion

The active NF-κB/IL-8 axis in the interaction between CRCs and SWs promoted tumor progression. Targeting the tumor-glial cells might be a novel anti-tumor therapy.

## Data availability statement

The data presented in the study are deposited in the Figshare repository, accession link: https://doi.org/10.6084/m9.figshare.21555090.v1

## Author contributions

SC designed the study, performed the experiments, analyzed the data, and wrote the paper. MC analyzed the data, critically revised the manuscript, and supervised all the experiments. All authors contributed to the article and approved the submitted version.

## Acknowledgments

The schematic diagram presented in this article were created with BioRender.com.

## Conflict of interest

The authors declare that the research was conducted in the absence of any commercial or financial relationships that could be construed as a potential conflict of interest.

## Publisher’s note

All claims expressed in this article are solely those of the authors and do not necessarily represent those of their affiliated organizations, or those of the publisher, the editors and the reviewers. Any product that may be evaluated in this article, or claim that may be made by its manufacturer, is not guaranteed or endorsed by the publisher.

## Supplementary material

Supplementary Figure 1 Schematic diagram of the co-culture system and the generation of CMs. **(A)** The process of SCs-CM collection. **(B)** The process of co-culture of SCs and HCT-116 or HT-29 cells and the collection of Schwann cells (Co-HCT-116)-CM or Schwann cells (Co-HT-29)-CM. SC, Schwann cell.
