# Supplementary figures and images for: Schwann cells promote the migration and invasion of colorectal cancer cells via the activated NF-κB/IL-8 axis in the tumor microenvironment
Source: Front Oncol. 2022 Nov 17;12:1026670. doi: 10.3389/fonc.2022.1026670 (PMC9714538; doi:10.3389/fonc.2022.1026670)

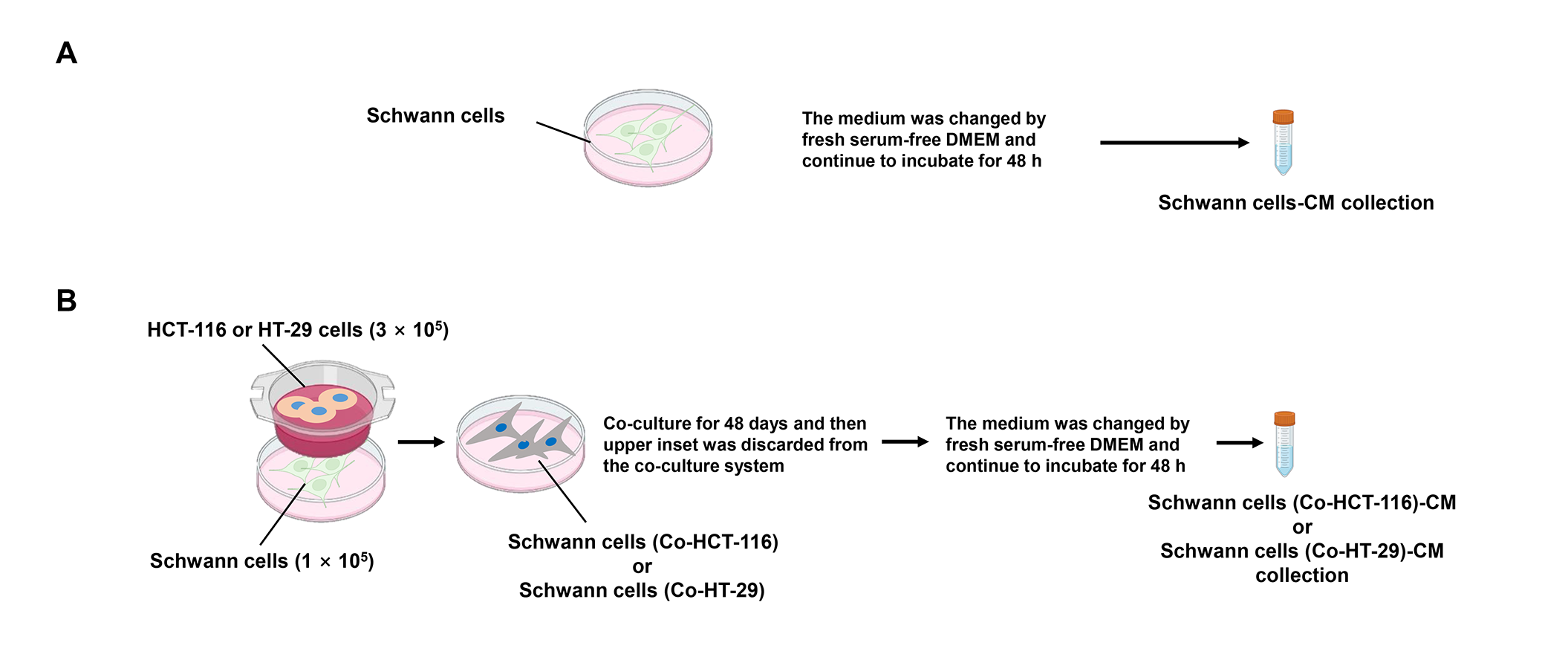

Supplement: Supplementary Figure 1 — Schematic diagram of the co-culture system and the generation of CMs. (A) The process of SCs-CM collection. (B) The process of co-culture of SCs and HCT-116 or HT-29 cells and the collection of Schwann cells (Co-HCT-116)-CM or Schwann cells (Co-HT-29)-CM. SC, Schwann cell. [file Image_1.tif]
